# The Roles of Long Non-Protein-Coding RNAs in Osteo-Adipogenic Lineage Commitment

**DOI:** 10.3390/ijms18061236

**Published:** 2017-06-09

**Authors:** Hirotaka Yoshioka, Yuji Yoshiko

**Affiliations:** Department of Calcified Tissue Biology, Hiroshima University Institute of Biomedical and Health Sciences, 734-8553 Hiroshima, Japan; hirotaka@hiroshima-u.ac.jp

**Keywords:** osteoblasts, adipocytes, transdifferentiation, epigenetics, non-protein-coding RNAs

## Abstract

Osteoblasts and adipocytes share a common mesenchymal progenitor in the bone marrow. This implies that a reciprocal relationship exists between osteogenic and adipogenic differentiation. Further, cells of osteoblast lineage transdifferentiate into adipocytes under some circumstances. Dysregulation of osteo-adipogenic fate-determination leads to bone diseases such as osteoporosis, accompanied by an increase in bone marrow adipose tissue. Thus, the fine-tuning of osteo-adipogenesis is necessary for bone homeostasis. Osteo-adipogenic progression is governed by a complex crosstalk of extrinsic signals, transcription factors, and epigenetic factors. Long non-protein-coding RNAs (lncRNAs) act in part as epigenetic regulators in a broad range of biological activities, such as chromatin organization, transcriptional regulation, post-translational modifications, and histone modification. In this review, we highlight the roles of epigenetic regulators, particularly lncRNAs, in the osteo-adipogenic lineage commitment of bone marrow mesenchymal stem cells and the adipogenic transdifferentiation of osteoblasts.

## 1. Introduction

Bone marrow mesenchymal stem cells (BMSCs) differentiate into multiple different cell types such as osteoblasts, adipocytes, and chondrocytes. BMSCs are also known to play an essential role in maintaining the hematopoietic stem cell niche by providing modulatory signals [1,2]. The bone marrow cavity is filled with active hematopoietic cells (red marrow) in newborns, and these cells are replaced with BMSC-derived adipocytes with age, resulting in the formation of marrow adipose tissue (MAT) [3]. MAT also accumulates in reaction to chemotherapy, glucocorticoid therapy, and radiotherapy [4]. Increased MAT is associated with bone pathologies including decreased cortical bone, low bone mineral density, decreased bone volume, and decreased bone formation rate [5]. Conversely, lipodystrophic mice that carry a hypomorphic allele of PPARγ, a master regulator of adipogenesis [6], show increased bone mass, with a reduction in bone marrow cavity [7]. The current understanding of the mechanism underlying MAT accumulation is that it occurs due to the increased expression of PPARγ which causes the commitment of BMSCs to the adipogenic lineage [8,9]. Thus, an inverse correlation exists between osteogenesis and adipogenesis, and the fine-tuning of the osteo-adipogenic fate-choice is crucial for bone and bone marrow health. Recent discoveries of non-protein-coding RNAs (ncRNAs) have opened an exciting new field of the epigenetic mechanisms involved in fate-choice decisions and differentiation processes in many different cell types [10,11]. For many years, ncRNAs had been considered as accessory components to aid protein activities. Non-coding *XIST* RNA was first identified to be directly involved in the inactivation of the X chromosome for dosage compensation [12]. Thereafter, multiple approaches, especially computational biology and RNomics, have led to the identification of numerous ncRNAs, such as microRNAs (miRNAs), lncRNAs, small nucleolar RNA, enhancer RNAs, and promoter-associated RNAs [10,11]. In this review, we highlight recent studies describing the epigenetic processes by which lncRNAs regulate osteogenic versus adipogenic differentiation in the bone marrow.

## 2. Epigenetics and ncRNAs

Lineage commitment of stem cells proceeds via multi-step, hierarchical processes under the control of transcription factors. With the onset of differentiation, stem cell plasticity becomes restricted due to acquisition of epigenetic modifications, such as DNA methylation and histone modifications, which impose cellular identity [13]. DNA methylation is a reversible biochemical process involving the addition of a methyl (–CH_3_) group to the C-5 position of cytosine in CpG dinucleotides [14]. This process is catalyzed by a family of DNA methyltransferases, including the maintenance methyltransferase DNMT1 and *de novo* methyltransferases DNMT3A and DNMT3B [14]. Histone modifications are also reversible processes where the N-terminal tails of histone proteins are subjected to post-translational modifications, such as acetylation, methylation, phosphorylation, and ubiquitylation [15]. These modifications are catalyzed by specific enzymes such as histone acetyltransferases, histone deacetylases (HDACs), histone methyltransferases, histone demethylases, and so on [15]. DNA methylation and histone modifications influence one another to form and stabilize higher-order chromatin structure [16].

**Figure 1:**
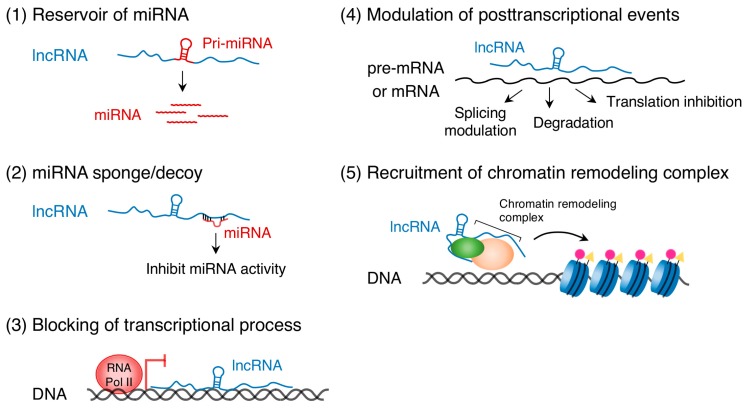
Long non-protein-coding RNAs (LncRNAs) play multiple roles in gene expression.

Recent studies have uncovered the important role of ncRNA transcripts in epigenetic gene regulation. ncRNAs are primarily classified into two categories, short ncRNAs (<200 nucleotides) and long ncRNAs (lncRNAs, >200 nucleotides) [10,11]. Short ncRNAs include miRNAs that are approximately 22 nucleotides in length and usually bind to 3′ untranslated regions of target mRNAs to repress transcription and translation (see [17,18,19,20] for review). The targets of miRNAs are not only protein-coding RNAs but also lncRNAs [21]. DNA methylation regulates the expression of miRNAs, such as miR-10a and miR-200, in human HCT116 cells [22]. In the opposite direction, the miR-290 family of miRNAs controls *de novo* DNA methylation in mouse embryonic stem cells [23]. LncRNAs regulate gene expression either in a *cis*- or *trans*-acting manner through multiple mechanisms (Figure 1): (1) serve as reservoirs of miRNAs [24,25], (2) act as miRNA sponges (competitive inhibitors of miRNAs) [26], (3) bind to complementary DNA sequences and form RNA-DNA triplex structures that can block the transcriptional process [27], (4) bind to pre-mRNA or mRNA and modulate posttranscriptional events such as splicing, degradation, and translation [28], and (5) recruit chromatin remodeling complexes to specific DNA loci [26,29,30]. (See other excellent reviews for more details, [31,32,33]) Thus, ncRNAs, DNA methylation, and histone modifications are mutually required to regulate their modifiers and chromatin signature, leading to a cascade of complex gene regulation events.

## 3. Epigenetics in Osteo-Adipogenesis

Lineage commitment of BMSCs is controlled by various extracellular cues such as transforming growth factor-β (TGF-β), WNT, Notch, Hedgehog, fibroblast growth factors, and insulin-like growth factors (see [34,35,36,37] for review). These molecules have dual roles in regulating osteoblastogenesis vs. adipogenesis. For instance, TGF-β acts as a bidirectional regulator of osteoblastogenesis by activating the SMAD-dependent pathways and the mitogen-activated protein kinase (MAPK)/extracellular signal-regulated kinase (ERK) pathways [37]. The former recruits HDAC4 and HDAC5 to repress expression of the osteoblast master regulator *RUNX2* in rat osteogenic ROS17/2.8 cells [38,39]. The latter phosphorylates RUNX2 to promote its transcriptional activity [40]. Overall, TGF-β acts on multiple steps of osteogenesis not only by promoting osteoprogenitor cell proliferation, early differentiation, and chemotaxis, but also by inhibiting osteoblast maturation, matrix mineralization, and osteocyte differentiation [37]. Concomitant with reduced adipogenesis in mouse 3T3-L1 and 3T3-F442A cells treated with TGF-β [41,42,43], transgenic mice overexpressing TGF-β show impaired development of adipose tissue [44]. This happens via the ERK pathway that phosphorylates PPARγ to inhibit its transcriptional activity and suppresses adipocyte differentiation [45,46,47]. In the case of WNT signaling, the β-catenin-dependent pathway activates osteoblastogenesis by directly stimulating *RUNX2* gene expression and by suppressing the adipogenic transcription factor genes *CEBPA* and *PPARG* in mouse stromal ST2 cells, as well as in 3T3-L1 cells [48,49,50,51]. KDM2A and KDM2B, demethylases of the trimethylation of histone H3 at lysine 4 (H3K4me3) and lysine 36 (H3K36me3), mediate the degradation of non-phosphorylated β-catenin in the nucleus of human embryonic kidney cells (HEK293T), suggesting that they have an inhibitory effect on osteogenesis [52]. Indeed, KDM2A/2B demethylases inhibit osteoblast differentiation by demethylating H3K4me3 and H3K36me3 in the promoter of *EREG* and *TFAP2A* in human mesenchymal stem cells (MSCs) [53,54]. On the other hand, β-catenin-independent WNT4/5A/5B signaling stimulates adipogenesis in 3T3-L1 cells [55,56,57]. Emerging evidence points to crosstalk between these signaling pathways and miRNAs [58]. MiR-181a targets genes involved in TGF-β signaling, such as *TGFBI* and *TGFBR1*, which are negative regulators of osteoblast differentiation [59]. MiR-346 promotes osteoblast differentiation via suppression of *GSK3B* to prevent the degradation of β-catenin in human BMSCs [60]. MiR-23a/b regulates the balance of osteo-adipogenic differentiation in human BMSCs by targeting *TMEM64*, which modulates WNT/β-catenin signaling [61,62]. These signaling pathways culminate in the activation of either RUNX2 or PPARγ [6,38]. RUNX2 and PPARγ reciprocally regulate one another during osteogenic and adipogenic differentiation, respectively, of BMSCs and adipose tissue-derived stem cells [47,63,64,65,66].

The cell-intrinsic epigenetic mechanisms underlying osteo-adipogenic lineage commitment have also been well studied. HDACs, key factors for bone formation [67] (see above), deacetylate N-terminal tails of histones and RUNX2 [68], thus mediating transcriptional repression and stability respectively. HDAC3, HDAC7, and HDAC8 interact with RUNX2 and act as a co-repressor of osteoblast-specific genes, such as *OCN*, *BSP*, and *RUNX2*, in mouse MC3T3-E1 and C2C12 cell lines, and rat BMSCs [69,70,71]. Osteochondral lineage specific *HDAC3* knockout mice show severe reductions in trabecular bone, bone formation rates, and osteoblast numbers, accompanied with increased MAT [72]. Mice lacking *HDAC8* in neural crest progenitor cells display ossification defects in frontal and interparietal bones [73]. Further studies are needed to resolve discrepancies between in vivo gene knockout studies and in vitro studies. HDACs also play a vital role in adipocyte differentiation. Inhibition of HDAC activity using sodium butyrate in 3T3-L1 cells induces adipogenic gene expression and adipocyte differentiation [74]. Preadipocytes isolated from *HDAC9*-knockout mice exhibit accelerated differentiation [75]. Besides, Jumonji C domain-containing histone lysine demethylases (e.g., the KDM family, NO66, RBP2, and PHF2) and SET domain-containing histone lysine methyltransferases (e.g., EZH2, SETDB1, and SETD8) are involved in osteo-adipogenic differentiation (see above for KDMs). For instance, KDM4B/6B demethylates the trimethylation of histone H3 at lysines 9 (H3K9me3) and 27 (H3K27me3), activating *RUNX2*, *SP7*, *DLX5*, *BMP*, and *HOX* genes. This results in enhanced osteoblast differentiation as demonstrated in MC3T3-E1 cells and human MSCs [76,77]. MiR-20a promotes adipocyte differentiation of ST2 cells by targeting *KDM6B* directly [78]. Preadipocytes isolated from mouse knockouts of *EZH2*, a gene that methylates H3K27, exhibit severe defects in adipogenic activity, concomitant with the derepression of *WNT1/6/10A/10B* genes, and with the activation of WNT/β-catenin signaling [79]. Deletion of *EZH2* in mesenchymal progenitor cells causes malformation of the growth plate, cranial suture, and the trabecular bone, accompanied by accumulation of MAT [80]. Thus, many epigenetic modifiers contribute to the balance of osteo-adipogenic differentiation (for more details see [81]).

## 4. Functional Roles of lncRNAs in TGF-β- and WNT-Dependent Osteo-Adipogenesis

As mentioned above, TGF-β and WNT signaling pathways are involved in osteo-adipogenesis [37,41,42,43,44,48,49,50,51,55,56,57]. These signaling pathways are modulated by lncRNAs and associated regulators, including miRNAs and histone modifiers (Table 1). The maternally expressed imprinted gene *H19* (2.3-kb lncRNA) acts as a primary miRNA precursor of miR-675. Its expression increases during osteoblast differentiation but decreases during adipocyte differentiation in human MSCs and BMSCs [24,25,26]. MiR-675 decreases the levels of mRNAs and proteins of TGF-β1 and class II histone deacetylases (HDAC4/5/6) in human MSCs [24]. HDAC4/5 repressor complexes are recruited by TGF-β1-activated SMAD2/3 to the *RUNX2* promoter region to suppress its transcription in ROS17/2.8 cells [39]. Consistent with these roles, miR-675 may indirectly increase *RUNX2* expression and osteoblast differentiation in human MSCs [24]. Overexpression of miR-675 in human BMSCs inhibits adipogenic differentiation through the downregulation of class II HDACs [25,82]. *H19* also acts as a miRNA sponge that captures miR-141, miR-22, miR-200a, and let-7 to inhibit their respective functions (in human BMSCs, HT-29 cells, and HEK293 cells [26,83,84]). MiR-141, miR-22, and miR-200a inactivate the WNT/β-catenin signaling pathway by suppressing β-catenin expression in several cell types, including human BMSCs, SGC7901 cells, and U251 cells [26,85]. MiR-141 and miR-200a suppress the expression of *DLX5*, a negative regulator of adipogenesis [86], with a resultant increase in osteoblast differentiation of MC3T3-E1 cells [87]. The role of let-7 in osteoblasts remains unknown, though this miRNA promotes adipogenesis in 3T3-L1 cells [88]. Thus, the regulatory effects of *H19* on osteo-adipogenesis are determined by its partner miRNAs.

The lncRNA *MEG3* has been shown to interact with chromatin and act as a repressive regulator in human breast cancer cells by RNA immunoprecipitation-coupled high-throughput sequencing [30]. *MEG3* interacts with the PRC2 complex and suppresses the expression of genes involved in the TGF-β pathway, such as *TGFB2*, *TGFBR1*, and *SMAD2*, in human breast cancer cells [30], thus inhibiting the SMAD-dependent signaling pathway. In human BMSCs, increasing the expression of *MEG3* activates *BMP4* transcription and promotes osteogenic differentiation [93]. The DNA methylation status of *MEG3* affects its expression in human leukocytes [94], and therefore, miR-29-mediated suppression of the DNA methyltransferases *DNMT1* and *DNMT3b* elevates the expression of *MEG3*, as demonstrated in hepatocellular cancer [95]. TGF-β1-mediated SMAD2/3 signaling negatively regulates the expression of miR-29 in mouse C2C12 myoblasts and human pancreatic stellate cells [96,97]. In human osteoblasts, canonical WNT signaling induces the expression of miR-29 which attenuates the effect of WNT signaling by targeting the WNT antagonists Dickkopf-1, Kremen2, and secreted frizzled related protein 2 [98]. It has also been shown that miR-29 promotes osteoblast differentiation by downregulating anti-osteogenic factors, such as *HDAC4*, *TGFB3*, *ACVR2A*, *CTNNBIP1*, and *DUSP2*, in MC3T3-E1 cells [99]. Thus, the *MEG3*-miR-29 regulatory circuitry may promote osteoblast differentiation, but its function on adipogenic differentiation remains unclear.

A recent, comprehensive survey of lncRNAs expressed differentially between bovine preadipocytes and differentiated adipocytes identified a novel lncRNA, *ADNCR*, that inhibits adipogenic differentiation by acting as a sponge of miR-204 [89]. Reduced expression of *ADNCR* with the onset of adipogenic differentiation increases free-functioning miR-204 that downregulates the target genes *RUNX2* and *SIRT1* [89,90]. Disruption of *RUNX2* in knockout mice shows maturation arrest of osteoblasts [38]. In addition, the suppression of *RUNX2* is indispensable for adipogenesis in 3T3-L1 cells [100]. SIRT1 is an adipogenic inhibitor; therefore, downregulation of *SIRT1* in C3H10T1/2 cells may promote adipogenic differentiation [101]. In addition, miR-204 suppresses WNT/β-catenin signaling by modulating *DVL3* expression, which promotes adipogenic differentiation of human adipose-derived MSCs [91].

In contrast to the above-mentioned lncRNAs, lncRNA *HOXA-AS3* has been identified as a promoter of adipogenesis [92]. The number of *HOXA-AS3* transcripts is found to increase gradually during adipogenesis of human MSCs. It interacts with EZH2 to recruit it to the *RUNX2* promoter and causes subsequent suppression of *RUNX2* and osteogenesis [92]. EZH2 catalyzes the methylation of H3K27 to form a repressive chromatin structure [81], and to repress WNT genes to facilitate adipogenesis [79]. *HOXA-AS3* may act on this process.

Thus, complex crosstalk among signaling molecules, ncRNAs, DNA methylases, and histone modifiers is crucial for achieving a balance between osteoblast and adipocyte lineage commitment.

## 5. Epigenetics in Osteo-Adipogenic Transdifferentiation

It was believed that differentiation was a unidirectional process and a terminal event to yield a stable cellular identity. However, as demonstrated by induced pluripotent stem cells, terminally differentiated cells can be experimentally reprogramed to function as pluripotent cells [102]. This conversion of cell fate is also observed in vivo during the healing process of tissues, such as intestine, liver, and so on [103]. Bone injury in zebrafish induces mature osteoblasts to dedifferentiate into osteoprogenitor cells that become a source of new reparative bone [104]. Mammalian mature osteoblasts appear not to behave as undifferentiated cells during bone healing processes [105]. However, rat calvaria-derived osteoblasts show a marked plasticity of phenotypes even at the maturation stage [106], suggesting that osteoblasts have the potential to switch their cell fates to other cell lineages by inductive extracellular cues. Indeed, we and other groups have shown fate shifts of osteoblast-lineage cells into adipocytes under certain conditions [107,108,109,110,111]. For instance, a subset of relatively mature rat osteoblasts expressing PPARγ become adipocytes when cultured with the synthetic PPARγ agonist rosiglitazone [108]. Loss of osteogenic WNT/β-catenin signaling in early osteoblast-lineage cells in vivo increases the number of adipocytes [109]. Recently, a subpopulation of CXC chemokine ligand 12-abundant reticular (CAR) cells that expresses mature osteoblast markers was identified [112]. CAR cells are mesenchymal progenitor cells having osteo-adipogenic potential [113]. Thus, a subpopulation of osteoblasts may transdifferentiate to the adipogenic lineage. These findings raise a question about how cell-intrinsic epigenetic programs participate in osteo-adipogenic transdifferentiation.

Epigenetic modifications undoubtedly serve as important regulators of cell plasticity and transdifferentiation potential [114]. Among multiple histone modifications, bivalent modifications of histone H3, through both active and repressive marking by H3K4me3 and H3K27me3, respectively, are thought to play a vital role in keeping genes poised for rapid activation in response to appropriate signals [115,116,117,118]. The presence of these modifications in a subset of genes is thought to correlate with cell plasticity [115,116,117,118]. Recent genome-wide analyses of histone modifications in mouse BMSCs reveal that the most highly expressed genes at stages of osteogenic commitment allow bivalent modifications before expressing them [119]. H3K27me3 removal activates specific genes that bestow osteogenic identities upon cells and reduce their plasticity [119]. Consistent with this and as mentioned above, histone demethylases of H3K27me3 such as KDM4B/5B/6A/6B play a pivotal role in osteogenic commitment [76,77,120,121]. Interestingly, the genes downregulated during osteogenesis rarely gain H3K27me3 [119], suggesting the absence of active mechanisms for repression during osteogenic differentiation. In contrast, EZH2 mediated H3K27me3 is vital for adipogenic lineage specification [120]. Genome-wide comparison of chromatin profiles during the osteo-adipogenic differentiation of BMSCs suggests a default preference for osteogenic differentiation [111]. Gene activation-associated histone modifications in BMSCs are also found in osteogenic differentiated cells, but not in adipogenic differentiated cells, indicating that osteoblasts encode innate plasticity through the epigenome [111]. Thus, the chromatin state of osteoblasts may allow them to switch their cell fate relatively easily.

Further studies are warranted to understand the mechanisms by which epigenetic modifiers, including ncRNAs, regulate the plasticity of osteoblasts. There is an increasing body of evidence suggesting that histone variants and nuclear architectures are involved in cell plasticity [114,122]. The epigenetic program may be even more complicated than has been discovered so far for determining osteo-adipogenic commitment.

## 6. Concluding Remarks and Outlook

Many epigenetic factors, including ncRNAs, play an important role in the fate choice of BMSCs and enable crosstalk among multiple signaling pathways. Genome-wide studies show aging-associated depletion of DNA methylation in humans and mice [123,124,125,126]. Since DNMT1 haploinsufficiency promotes age-related health problems, including decreased bone mineral density [127], the genome-wide age-related decrease in DNA methylation may be attributed to a reduction of DNMT1 activity [128]. Similarly, aging-associated alterations are observed in the context of chromatin organization [129]. The histone deacetylase SIRT1 is known to function as a longevity factor, and its deficiency leads to premature aging, in parallel with global enrichment of acetylation at lysine 16 of histone H4 [130]. *SIRT1* osteoblast-specific knockout mice show a significant decrease in trabecular bone volume and trabecular thickness [131]. Downregulation of HDAC1 and HDAC2 is also observed during senescence of human MSCs, followed by downregulation of EZH2 and upregulation of KDM6B [132]. These age-related alterations of histone-modifying enzymes contribute to perturbation in heterochromatin structure with aging [128]. Changes in the expression of ncRNAs are also believed to be associated with age-associated diseases and degenerative diseases [133,134]. Cell identity is mediated by epigenetics, so epigenetic aberrations may affect the plasticity of both stem and differentiated cells [135]. This idea is supported by recent evidence that biological senescence and tissue injury promotes epigenetic cellular reprogramming in vivo [136]. Therefore, age-related increases in PPARγ and/or adipogenic miRNA expression may affect not only the fate-choice decisions of BMSCs, but also the plasticity of the osteoblast population [8,137]. Whether osteoblasts convert their fates into adipocytes in vivo still remains controversial. Lineage tracing studies demonstrate the potential of cells in the mesenchymal lineage to transdifferentiate, for instance, hypertrophic chondrocytes transdifferentiate into osteoblasts during the endochondral bone formation and fracture-healing processes [138,139]. Forced expression of transcription factors and ncRNAs bestows the ability upon cells to directly convert from one cell type to another in vivo [140,141]. Given these observations, epigenetic alterations with age may induce transdifferentiation of osteoblasts into adipocytes, which may explain age-associated MAT accumulation. We believe that functional studies of ncRNAs and their interacting partners during osteo-adipogenesis may open new prospects for the prevention of MAT accumulation and bone loss in the future.

## Figures and Tables

**Table 1:** LncRNAs and osteo-adipogenic fate decision.

LncRNAs	Expression Profiles	Functions	Effector Molecules	Target Genes	References
*H19*	Osteogenic ↑	miRNA precursor	miR-675	*TGFB1, HDAC4, HDAC5, HAC6*	[24,25]
	Adipogenic ↓	miRNA sponge	miR-200a, miR-141, miR-22, let-7	*DLX5, CTNNB1*	[26,83,84,85,87]
*MEG3*	Osteogenic ↑	protein recruiter	PRC2	*TGFB2, TGFBR1, SMAD2*	[30]
	Adipogenic ↓				
*ADNCR*	Osteogenic ?	miRNA sponge	miR-204	*RUNX2, SIRT1, DVL3*	[89,90,91]
	Adipogenic ↓				
*HOXA-AS3*	Osteogenic →	protein recruiter	EZH2	*RUNX2*	[92]
	Adipogenic ↑				

↑, upregulation; ↓, downregulation; →, no change in expression; ?, not determined.

## Abbreviations

lncRNAs	Long non-coding RNAs
BMSCs	Bone marrow mesenchymal stem cells
MAT	Marrow adipose tissue
ncRNAs	Non-protein-coding RNAs
miRNAs	MicroRNAs
HDACs	Histone deacetylases
TGF-β	Transforming growth factor-β
MAPK	Mitogen-activated protein kinase
ERK	Extracellular signal-regulated kinase
H3K4me3	Trimethylation of histone H3 at lysine 4
H3K36me3	Trimethylation of histone H3 at lysine 36
MSCs	Mesenchymal stem cells
H3K9me3	Trimethylation of histone H3 at lysine 9
H3K27me3	Trimethylation of histone H3 at lysine 27
CAR cells	CXC chemokine ligand 12-abundant reticular cells
